# Genetic variations in triple-negative breast cancers undergoing neo-adjuvant chemotherapy

**DOI:** 10.20517/cdr.2019.44

**Published:** 2019-09-19

**Authors:** Miki Mori, Tomoko Watanabe, Sadako Akashi-Tanaka, Kumiko Ueda, Reiko Makino, Yuko Hirota, Seigo Nakamura

**Affiliations:** ^1^Department of Breast Surgical Oncology, Showa University, Shinagawaku, Tokyo 142-8666, Japan.; ^2^Clinical Research Laboratory, Showa University, Shinagawaku, Tokyo 142-8666, Japan.; ^3^Department of Pathology, Showa University, Shinagawaku, Tokyo 142-8666, Japan.

**Keywords:** Triple negative breast cancer, next generation sequence, TP53 and neo adjuvant chemotherapy

## Abstract

**Aim:** Triple negative breast cancer (TNBC) is known as aggressive subtype and have no identified targeted therapies. We examined the relationship of neoadjuvant chemotherapy response to genetic variations of TNBC.

**Methods:** The tumors used in this study were collected from Showa University Hospital, Japan. Thirteen formalin-fixed paraffin-embedded tumors from Japanese TNBC patients who underwent neoadjuvant chemotherapy were used for analysis. Of these, eight surgically resected tumors showed progressive disease and/or recurrence after treatment (PD/REC), and biopsy tissues from five patients showing pathological complete response (pCR) were analyzed. DNA extracted from tissue sample were analyzed. The Miseq system and Trusight Tumor Sequence panel kit were used to sequence 174 amplicons over 82 exons of 26 cancer-related genes to identify genetic mutations.

**Results:** Seven somatic non-synonymous variants were detected in three genes (*FOXL2*, *PIK3CA*, and *TP53*) in all five pCR patients, and six somatic non-synonymous variants in two genes (*PTEN* and *TP53*) were detected in six of eight PD/REC patients. Eight of 13 TNBC tumors were found to have TP53 pathogenic variants, in both pCR and PD/REC cases.

**Conclusion:** Although *TP53* variation was detected in both pCR and PD/REC cases, each location and type of the variant were different. We could not identify genetic mutations associated with chemotherapy response and recurrence.

## Introduction

Triple-negative breast cancer (TNBC) is defined as any breast cancer lacking estrogen receptor, progesterone receptor, and human epidermal growth factor receptor 2 expression. It accounts for approximately 15%-20% of all breast cancers^[1,2]^. TNBC is reported to behave more aggressively and is associated with a worse survival than other types of breast cancer^[3,4]^. Patients with TNBC have no identified targeted therapies. They are currently treated by surgery and chemotherapy alone^[1]^.

Triple-negative subtypes are more sensitive to neo-adjuvant chemotherapy than luminal breast cancers^[5-7]^. Patients with TNBC have increased pathologic complete response (pCR) rates compared with non-TNBC patients, and those with pCR have excellent survival regardless of subtype^[5-7]^. However, patients with progressive disease after neo-adjuvant chemotherapy have a significantly worse prognosis if they have TNBC compared with other subtypes^[5,7]^. About 70%-80% of patients of TNBC fail to respond to neoadjuvant chemotherapy (NAC)^[5-7]^, but the molecular mechanism underlying differences in responders and non-responders is unclear. It is also poorly understood which genes and pathways are related to treatment response in breast tumors.

Herein, we performed an exploratory study using next generation sequencing to investigate the dynamics of tumor gene mutations in TNBC patients receiving NAC.

## Methods

### Ethical approval

This study was approved (number 1589) by the Institutional Review Board of Showa University, Japan. Written informed consent was received from the patients. If informed consent could not be received for the death of patients, the candidate opted out on the homepage of the hospital.

### Patient information

The tumors analyzed in this study were collected from Showa University Hospital, Japan. Ninety four patients revealed triple negative subtype of 1111 patients of primary breast cancer surgery between 2011 and 2013. Of these 94 patients, 33 underwent neo adjuvant chemotherapy prior to surgery. Ten patients achieved pCR and remaining 23 patients had residual disease. Four patients revealed clinically progressive disease during neo adjuvant chemotherapy. Taxane followed by anthracycline based regimens were applied as standard neo-adjuvant chemotherapy. Two patients who revealed progressive disease during first taxane regimen dropped out of neo-adjuvant chemotherapy then planned surgery without following anthracycline chemotherapy. Seven patients of 23 patients who had residual disease including 3 progressive disease revealed recurrence in the same period. In total, there were 8 patients with progressive disease and/or recurrence (PD/REC). Five of 10 pCR cases underwent pretreatment biopsy in Showa University Hospital and able to obtain the sample. These 8 PD/REC cases and 5 pCR cases were analyzed. Formalin-fixed paraffin-embedded (FFPE) samples from pretreatment biopsy of PD/REC and pCR cases and surgical specimen of PD/REC cases were obtained. Clinical and pathologic information was obtained from medical chart including the information of the results of both germline BRCA1/2 testing and BRNAness. The concept of “BRCAness” is that sporadic basal-like breast cancers resemble BRCA1 mutated cancers. A set of 34 MLPA probes was used to identify BRCAness^[8]^. American Joint Committee on Cancer/tumor-node-metastasis cancer staging was assessed.

### Preparation of genomic DNA

Ten slices of 10 μm-thick FFPE tissues were used for genomic DNA extraction. Tissues were de-paraffinized with xylene, then DNA was extracted using the QIAamPD/recNA FFPE Tissue Kit (Qiagen, Venlo, Netherlands) following the manufacturer’s instructions.

Briefly, each deparaffinization solution, specific buffers and proteinase was added to destruct cells while centrifuging. After incubation, ethanol was added to precipitate DNA. After transferring the entire lysate to purification column, the centrifugation was repeated. Finally, elution buffer was added to elute DNA in the column.

### Target genes

The TruSight Tumor Sequencing panel (Illumina, San Diego, California, USA, #FC-130-2001) was used to detect genetic variations in tumors using NGS. This panel covered 174 amplicons over 82 exons of 26 cancer-related genes. The following genes encode kinases: *AKT1*, *ALK*, *BRAF*, *EGFR*, *ERBB2*, *FGFR2*, *KIT*, *MAP2K1*, *MET*, *PD/RECGFRA*, *PIK3CA*, *SRC*, and *STK11*, while the remainder do not: *APC*, *CDH1*, *CTNNB1*, *FBXW7*, *FOXL2*, *GNAQ*, *GNAS*, *KRAS*, *MSH6*, *NRAS*, *PTEN*, *SMAD4*, and *TP53*.

### Sequencing

The quality of the genomic DNA purified from FFPE tissues was checked by quantitative PCR using Quality Control Template (Illumina) and iQ^TM^ SYBR® Green Supermix (BioRad). Samples were required to fulfill the criteria ΔCt < 5, which was typically 30-300 ng depending on the DNA quality. However, ΔCt = 12 biopsy specimens were also included in the analysis. Genomic DNA hybridization-based enrichment was performed using the TruSight Tumor Sequence panel kit according to the manufacturer’s protocol. Pooled amplicons were end-repaired then underwent adapter ligation. The purified library was quantified using the Qubit® fluorometer with the Qubit® dsDNA BR assay kit. Finally, the captured DNA was subjected to 121-bp paired-end read sequencing on the MiSeq system (Illumina).

### Strategies for the analysis of genetic variations

Sequence reads were exported into FASTQ format files. The alignment of pair-end sequencing reads to the reference genome was performed using a banded Smith-Waterman method with the Amplicon-DS workflow software plug-in for MiSeq Reporter. Human Genome version 19 (UCSC) was used as the alignment reference. The identification of sequencing variants was performed using the Illumina Somatic Variant Caller algorithm. The called SNPs/indels were annotated using COSMIC (somatic mutation information), the IARC TP53 database (human TP53 gene variations related to cancer)^[9]^, and other databases of genetic variations. Database was aggregated into the Illumina VariantStudio application. Additionally, NM_000179.2:c.3254delC and NM_000179.2:c.3253_3254insC in *MSH6* were eliminated as known artifact variants. Statistical analysis was performed with Fisher’s exact test.

## Results

### Clinical features of the patients

The clinical features of the patients are summarized in the Table 1. Patients had an average age of 49.6 years. Seven cases underwent *BRCA1* and *BRCA2* germline mutation testing. *BRCA1/2* gene testing are not covered by national healthcare insurance, so it was performed to limited patients based on age, family history by self-pay. Seven PD/REC cases, including three patients with germline mutation showed BRCAness. One pCR case also showed BRCAness.

**Table 1:** Clinical features of the patients

	pCR	PD/REC
Age, year		
Median (range)	56 (40-72)	46 (27-68)
Clinical T-stage at diagnosis		
T1	1	0
T2	2	7
T3	2	1
T4	0	0
Clinical N-stage at diagnosis		
N0	2	5
N1	1	3
N2	2	0
N3	0	0
Stage at diagnosis		
I	1	0
II	1	7
III	3	1
IV	0	0
Nuclear grade		
1	0	0
2	1	3
3	4	4
Not available	0	1
Ki-67		
< 20%	0	0
≥ 20%	5	7
Not available	0	1
Histology		
Ductal	4	8
Lobular	0	0
Others	1	0
Subtype defined by IHC		
EGFR(+), CK5/6(+)	0	2
EGFR(+), CK5/6(-)	0	1
EGFR(-), CK5/6(+)	1	1
EGFR(-), CK5/6(-)	2	3
Not tested	2	1
BRCA mutation		
Deleterious	0	3
Negative	2	2
Not tested	3	3
BRCAness		
Yes	1	7
No	1	0
Not analyzed	3	1
Neo-adjuvant treatment		
Anthracycline + Taxane	4	6
Taxane + Other	0	2
Other	1	0
Surgery type		
Mastectomy	1	6
Lumpectomy	4	2
Sentinel Node Biopsy only	2	4
Axillary lymph node dissection	3	4
Yeild of pathological T-stage		
T0	5	0
T1	0	4
T2	0	3
T3	0	1
T4	0	0
Yeild of pathological N-stage		
N0	5	4
N1	0	4
N2	0	0
N3	0	0
Yeild of pathological stage		
I	0	3
II	0	4
III	0	1
IV	0	0
Adjuvant treatment		
No	5	4
FEC	0	1
Gemcitabine + Carboplatin	0	3
Recurrence		
Yes	0	7
No	5	1
Radiation		
Yes	4	4
No	1	4
Death		
Yes	0	3
No	5	5
Average DFS (months)	25	17
Average observation period (months)	34	40

pCR: pathological complete response; PD/REC: progressive disease and/or recurrence; IHC: immunohistochemistry; DFS: disease free survival; FEC: fluorouracil epirubicin cyclophosphamide

### Genetic variations in tumors showed PD/REC and pCR

The genetic variations after filtering out synonymous variants and known artifacts are shown in the Table 2. Three biopsy samples of 8 PD/REC cases were not available for the analysis. In PD/REC cases, we found ten non-synonymous variants were detected in the coding regions of two genes in 5 biopsy tissues before NAC, while 14 non-synonymous variants were detected in the coding regions of three genes in 8 surgical specimens after NAC. In 5 pCR cases, ten non-synonymous variants were detected in the coding regions of four genes. Of these, variants *TP53* Val31Ile and Pro72Arg, *STK11* Phe354Leu, and *MET* Asn375Ser are known polymorphisms (NIH dbSNP: rs33917957, rs1042522, rs59912467 and rs33917957, respectively).

**Table 2:** Genetic vatiations detected in pCR/PD groups

Sample ID	Biopsy samples before NAC			Surgical specimens after NAC		
	Gene	Pathological variations	Polymorphisms	Gene	Pathological variations	Polymorphisms
Genetic variations in PD/REC patients						
6	*TP53*		p.Val31Ile	*TP53*		p.Val31Ile
	*TP53*		p.Pro72Arg	*TP53*		p.Pro72Arg
	*STK11*		p.Phe354Leu	*STK11*		p.Phe354Leu
7	*TP53*		p.Pro72Arg	*TP53*		p.Pro72Arg
				*PTEN*	p.Ser179Valfs*8	
8	*TP53*	p.Val272Met	p.Pro72Arg	*TP53*	p.Val272Met	p.Pro72Arg
9	n.a.			*TP53*	p.Arg333Valfs*12	
10	n.a.			*TP53*		p.Pro72Arg
11	*TP53*	p.Cys275Trp	p.Pro72Arg	*TP53*	p.Cys275Trp	p.Pro72Arg
12	n.a.			*TP53*	p.Val216Met	
13	*TP53*	p.Ile195Thr	p.Pro72Arg	*TP53*	p.Ile195Thr	p.Pro72Arg
Genetic variations in pCR patients						
1	*TP53*	p.Leu111Phefs*40				
2	*TP53*	p.Val157Phe	p.Pro72Arg			
	*Met*		p.Asn375Ser			
3	*TP53*	p.Cys141Tyr	p.Pro72Arg			
4	*TP53*	p.Arg342*	p.Pro72Arg			
	*FOXL2*	p.Leu130Gln				
5	*PIK3CA*	p.Arg1023Glnfs*4				

NAC: neoadjuvant chemotherapy; pCR: pathological complete response; PD/REC: progressive disease and/or recurrence; n.a.: not analyzed

As a result, total of 3 somatic mutations in one gene (*TP53*) and six somatic mutations in two genes (*TP53* and *PTEN*) were detected in biopsy samples (3/5 cases) and surgical specimens of PD/REC patients (6/8 cases), respectively. Somatic mutations were detected in three genes *TP53*, *FOXL2* and *PIK3CA* in pCR patients (5/5 cases).

*TP53* was the most frequently mutated gene (9 of 13, 69.2% in total, 5 of 8, 62.5% in PD/REC cases and 4 of 5, 80% in pCR cases) (*P* = 0.50). Other pathogenic variants detected at a lower frequency were in genes *PIK3CA* (*n* = 1), *PTEN* (*n* = 1) and *FOXL2* (*n* = 1). In both PD/REC and CR, unique pathogenic variants were detected in *TP53* and other genes. As shown in the Table 3, the *TP53* Pro72Arg polymorphism was detected in both pCR and PD/REC cases.

**Table 3:** Genotype of the TP53 Pro72Arg polymorphism

Sample ID	Genotype	Amino acid	Genotype	Respose to chemothrapy
1	C/C	Pro/Pro	-	pCR
2	C/G	Pro/Arg	Heterozygote	pCR
3	G/G	Arg/Arg	Homozygote	pCR
4	G/G	Arg/Arg	Homozygote	pCR
5	C/C	Pro/Pro	-	pCR
6	C/G	Pro/Arg	Heterozygote	PD/REC
7	C/G	Pro/Arg	Heterozygote	PD/REC
8	G/G	Arg/Arg	Homozygote	PD/REC
9	C/C	Pro/Pro	-	PD/REC
10	C/G	Pro/Arg	Heterozygote	PD/REC
11	G/G	Arg/Arg	Homozygote	PD/REC
12	C/C	Pro/Pro	-	PD/REC
13	C/C	Pro/Pro	-	PD/REC

pCR: pathological complete response; PD/REC: progressive disease and/or recurrence

Next, we compared the effect of chemotherapy in five matched cases showed PD/REC. In patient 7, the *PTEN* Ser179Valfs*8 mutation was detected after NAC, but it was seen before NAC at low frequency on one strand (2%). Other genetic variations (*TP53* and *STK11*) were observed both pre- and post-treatment.

## Discussion

In this study, we used NGS to analyze 26 genes of 13 TNBC tumors receiving NAC. This is the first report to determine genetic variations in Japanese TNBC patients who showed pCR and PD/REC as a result of NAC. *TP53* pathogenic variants were detected in both pCR and PD/REC cases at frequencies similar to the high rates previously reported. *TP53* genetic variations in pCR cases might be associated with sensitivity to chemotherapy, and other *TP53* variants in PD/REC cases might affect resistance to chemotherapy. Thus, sensitivity to chemotherapy may vary by the location and type of somatic *TP53* variants. However, we could not confirm whether these genetic alterations found in this study were responsible for the chemo-sensitivity and recurrence because the number of patients are too small.

The numbers of genes and cases assessed were limited in this study. However, the frequency of genetic variations detected in TNBC did not differ greatly from those in previous studies. Lips *et al.*^[10]^ analyzed 1977 genes in 56 pre-treatment TNBC biopsies of NAC responders and non-responders as well as matched normal DNA. They reported that most mutations were in *TP53*, *TTN*, and *PIK3CA* (55%, 14%, and 9%, respectively). No recurrent mutations were associated with chemotherapy response or relapse^[10]^. Balko *et al.*^[11]^ examined residual disease in 74 clinically defined TNBCs after NAC, which included NGS performed on 20 matched pretreatment biopsies. Targeted NGS was carried out of 3320 exons from 182 oncogenes and tumor suppressors as well as 37 introns of 14 genes frequently rearranged in cancer. The frequency of each mutation was fewer than 5% of samples. Alterations in *TP53* were identified in 72 of 81 samples (89%), which was a similar rate to that observed in other studies of basal-like breast cancer or TNBC, and The Cancer Genome Atlas dataset (~85%)^[11]^. Roy-Chowdhuri *et al.*^[12]^ reported that a triple-negative group (*n* = 77) showed mutations in 15 of 46 genes tested, with *TP53* showing the highest mutation frequency (*n* = 48/77, 62%), followed by *PIK3CA* (*n* = 13/77, 17%), *APC*, *RET*, *SMAD4* (*n* = 2, 3%), *AKT1*, *ATM*, *BRAF*, *FGFR1*, *HRAS*, *JAK3*, *MET*, *SRC*, *PTEN*, and *STK11* (*n* = 1, 1%)^[12]^. From these previous reports, it is difficult to conclude that the varied and sparse genetic alterations seen in the present study caused differences between responders and non-responders toward chemotherapy.

*TP53* mutations in breast cancer have previously been reported to be associated with worse prognosis^[13,14]^. Ooe *et al.*^[15]^ investigated the relationship between the p53 mutation status and response to docetaxel in breast cancers. They found that the response rate of patients with p53-mutated tumors (44%) was lower than that of those with wild-type tumors (62%). In addition, the *TP53* p.Pro72Arg polymorphism was reported association with platinum-based chemotherapy response in non-small cell lung cancer^[16]^. Platinum-based chemotherapy was only administered to one case in our study, so no response difference was detected between heterozygous and homozygous variants in the pCR or PD/REC group. However, the same genetic variations of *TP53* (p.V157F and p.Cys141Tyr) detected in PD/REC samples in previous study^[11]^ were also observed in pCR samples in our study.

As a cohort, it is representative of Japanese TNBC, but the number of patients and samples was too low to confirm relevant relationship with genetic variants. This preliminary study should be followed up with a higher number of patients and selected genes in future.

In this study, 8 of 13 TNBC tumors were found to have *TP53* pathogenic variants, in both pCR and PD/REC cases. *TP53* variations were detected at similarly high rates as previously reported and may be associated with sensitivity or resistance to chemotherapy depending on the location and type of the variant. If the effect of these genetic variations on the induction of apoptosis could be determined, this might enable the mechanism of the NAC chemotherapy response to be understood. Our results provide insights into potentially actionable variants for targeted therapeutic options in TNBC.

## Declarations

### Copyright

© The Author(s) 2019.

## References

[1] Iwase H, Kurebayashi J, Tsuda H, Ohta T, Kurosumi M (2010). Clinicopathological analyses of triple negative breast cancer using surveillance data from the registration committee of the Japanese Breast Cancer Society.. Breast Cancer.

[2] Foulkes WD, Smith IE, Reis-Filho (2010). Triple-negative breast cancer.. N Engl J Med.

[3] Dent R, Trudeau M, Pritchard KI, Hanna WM, Kahn HK (2007). Triple-negative breast cancer: clinical features and patterns of recurrence.. Clin Cancer Res.

[4] Lin NU, Vanderplas A, Hughes ME, Theriault RL, Edge SB (2012). Clinicopathologic features, patterns of recurrence, and survival among women with triple-negative breast cancer in the National Comprehensive Cancer Network.. Cancer.

[5] Luangdilok S, Samarnthai N, Korphaisarn K (2014). Association between pathological complete response and outcome following neoadjuvant chemotherapy in locally advanced breast cancer patients.. J Breast Cancer.

[6] Liedtke C, Mazouni C, Hess KR, André F, Tordai A (2008). Response to neoadjuvant therapy and long-term survival in patients with triple-negative breast cancer.. J Clin Oncol.

[7] Carey LA, Dees EC, Sawyer L, Gatti L, Moore DT (2007). The triple negative paradox: primary tumor chemosensitivity of breast cancer subtypes.. Clin Cancer Res.

[8] Lips EH, Laddach N, Savola SP, Vollebergh MA, Oonk AM (2011). Quantitative copy number analysis by Multiplex Ligation-dependent Probe Amplification (MLPA) of BRCA1-associated breast cancer regions identifies BRCAness.. Breast Cancer Res.

[9] Petitjean A, Mathe E, Kato S, Ishioka C, Tavtigian SV (2007). Impact of mutant p53 functional properties on TP53 mutation patterns and tumor phenotype: lessons from recent developments in the IARC TP53 database.. Hum Mutat.

[10] Lips EH, Michaut M, Hoogstraat M, Mulder L, Besselink NJ (2015). Next generation sequencing of triple negative breast cancer to find predictors for chemotherapy response.. Breast Cancer Res.

[11] Balko JM, Giltnane JM, Wang K, Schwarz LJ, Young CD (2014). Molecular profiling of the residual disease of triple-negative breast cancers after neoadjuvant chemotherapy identifies actionable therapeutic targets.. Cancer Discov.

[12] Roy-Chowdhuri S, de Melo Gagliato D, Routbort MJ, Patel KP, Singh RR (2015). Multigene clinical mutational profiling of breast carcinoma using next-generation sequencing.. Am J Clin Pathol.

[13] Olivier M, Langerød A, Carrieri P, Bergh J, Klaar S (2006). The clinical value of TP53 gene mutations in 1794 patients with breast cancer.. Clin Cancer Res.

[14] Langerød A, Zhao H, Borgan Ø, Nesland JM, Bukholm IR (2007). TP53 mutation status and gene expression profiles are powerful powerful prognositic markers of breast cancer.. Breast Cancer Res.

[15] Ooe A, Kato K, Noguchi S (2007). Possible involvement of CCT5, RGS3, and YKT6 genes up-regulated in p53-mutated tumors in resistance to docetaxel in human breast cancers.. Breast Cancer Res Treat.

[16] Shiraishi K, Kohno T, Tanai C, Goto Y, Kuchiba A (2010). Association of DNA repair gene polymorphisms with response to platinum-based doublet chemotherapy in patients with non-small-cell lung cancer.. J Clin Oncol.

